# Accuracy of Self-Reported Height and Weight in Children Aged 6 to11 Years

**DOI:** 10.5888/pcd9.120021

**Published:** 2012-06-28

**Authors:** Jimikaye Beck, Christine A. Schaefer, Heidi Nace, Alana D. Steffen, Claudio Nigg, Lois Brink, James O. Hill, Raymond C. Browning

**Affiliations:** Author Affiliations: Jimikaye Beck, Christine A. Schaefer, Heidi Nace, Colorado State University, Fort Collins, Colorado; Alana D. Steffen, Claudio Nigg, University of Hawaii, Honolulu, Hawaii; Lois Brink, James O. Hill, University of Colorado Denver, Denver, Colorado.

## Abstract

The purpose of this study was to determine the ability of first-, third-, and fifth-graders to accurately self-report height and weight. Self-reported and measured values for height and weight were recorded for 487 students. The ability to self-report a reasonable value for height and weight improved with grade level, but children in all 3 grade levels significantly underreported their height and weight. Only fifth-graders accurately self-reported their weight; therefore, using self-reported height and weight to determine the prevalence of overweight and obesity for elementary school–aged children is not recommended.

## Objective

Self-reported height and weight measures are among the most common methods used to determine obesity prevalence, and ample research on adults’ and adolescents’ ability to self-report exists (1–3); however, research among elementary school–aged children is limited (4,5). Understanding the accuracy of self-report is necessary to determine the appropriateness of using such measures to determine obesity prevalence (6). The purpose of this study was to determine the ability of elementary school–aged students to self-report height and weight. We hypothesized that the accuracy of self-reporting improves with age and that children underestimate their weight, leading to inaccurate estimations of overweight and obesity prevalence.

## Methods

Participants were part of a large, multischool intervention that examined the effects of the playground environment and an educational intervention on physical activity levels in elementary school students (IPLAY study). Data were collected from 487 randomly selected first-, third-, and fifth-graders who attended 1 of 8 Denver, Colorado, metro-area elementary schools in April and May of 2010 and 2011. The Institutional Review Board for Human Subjects Research at Colorado State University approved this study. Self-reported height and weight were obtained verbally using the questions “How tall do you think you are?” and “How much do you think you weigh?” Participants were encouraged to guess if they were uncertain of their height or weight, and they were given time to respond. Height measurements were taken to the nearest 0.001 m and were recorded using a tape measure secured to the wall and a bubble level, as children stood with their heels and back against the wall. Weight measurements were recorded to the nearest 0.1 kg using a digital scale (Health o meter, Model 349KLX, Jarden Corporation, Rye, New York). Children were weighed and measured while wearing indoor clothing and shoes. Measured and self-reported values were judged “reasonable” or “unreasonable” on the basis of biologically implausible values, determined by using Centers for Disease Control and Prevention’s body mass index (BMI) percentile-for-age SAS macro (SAS Institute, Inc, Cary, North Carolina), and World Health Organization outlier cutoffs (7). Mean measured and self-reported height and weight, as well as the correlation between these variables for each grade level, were compared using paired *t* tests and linear regression, respectively. Self-reported BMI was calculated only for students who reported reasonable values for both height and weight. Measured BMI was calculated for all students with measured values for both height and weight. Prevalence of overweight was determined according to BMI-for-age *z* scores (8).

## Results

Mean (standard deviation, SD) ages of participants were 7.1 (0.5) for first-graders, 9.1 (0.4) for third-graders, and 11.2 (0.5) for fifth-graders. Mean (SD) heights were 1.24 (0.06) meters for first-graders, 1.37 (0.06) meters for third-graders, and 1.49 (0.07) meters for fifth-graders. Mean (SD) weights were 26.1 (6.0) kilograms for first-graders, 33.6 (8.3) kilograms for third-graders, and 44.4 (11.5) kilograms for fifth-graders. Average BMI percentile-for-age values were 59.5 (28.8) for first-graders, 59.5 (30.5) for third-graders, and 62.4 (31.7) for fifth-graders. The number of participants classified as obese was 144 (25.6% of first-graders, 28.5% of third-graders, and 34.5% of fifth-graders). 

The percentage of students who reported a reasonable height or weight ranged from 20% (first grade, height) to 92% (fifth grade, weight) (Table). In general, self-report ability was better in older children and when self-reporting weight. After removing the data on children who did not report reasonable height and weight values, we found that children in all 3 grade levels significantly underreported their height and weight (Table). Correlation coefficients improved with age and were greater when reporting weight versus height. The accuracy of self-reported weight decreased as BMI *z* score increased (Figure). Based on self-report, 35% of fifth-graders in our study were classified as overweight or obese, whereas measured values indicated that only 29% of fifth-graders who reasonably reported were overweight or obese.

**Table:** Percentage of Children Self-Reporting Reasonable Values for Height and Weight and Difference Between Measured and Self-Reported Height, Weight, and Body Mass Index, Denver, Colorado, 2010–2011^a^

Grade	Height				Weight				BMI		

	%	SR, m	D, m	*r* ^2^	%	SR, kg	D, kg	*r* ^2^	%	D, kg/m^2^	*r* ^2^
First (n = 21)	20	1.169 (0.019)	0.079^b^	0.22	48	22.6 (0.7)	3.30^b^	0.60	10	6.45^b^	0.67
Third (n = 61)	44	1.325 (0.015)	0.063^b^	0.38	78	30.7 (0.7)	2.82^b^	0.75	32	−1.97^b^	0.59
Fifth (n = 123)	78	1.441 (0.011)	0.050^b^	0.49	92	39.8 (0.9)	3.71^b^	0.86	65	2.91^b^	0.64

Abbreviations: BMI, body mass index; SR, self-reported; D, difference; SD, standard deviation.

^a^ The percentages represent children who reported reasonable values for height and weight. Difference is the difference between the measured and self-reported values. Pearson correlation coefficients are the difference between measured and self-reported values for each variable. Self-reported values are reported as mean (SD).

^b^ Difference between measured and self-reported value significant at *P* < .05.

**Figure:**
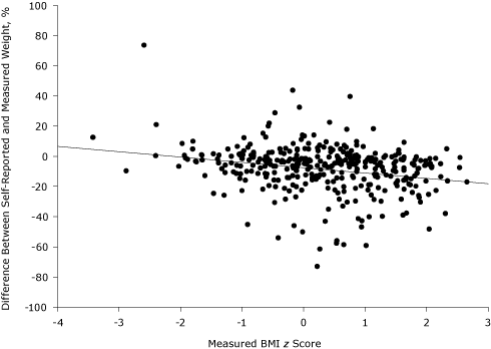
Measured BMI *z* score and percentage difference between self-reported and measured weight. Data on the scatter plot demonstrate that accuracy of self-reported weight decreased as BMI *z* score increased. A negative value for the percentage difference between weight indicates an underestimation of weight. For example, a child who weighs 30 kg but self-reported 27 kg is indicated by a −10% difference between the measured and self-reported weight. Solid line represents the linear regression fit. Weight % difference = −0.0757 − (0.0353 × BMIz); *r* ^2^ = 0.061; *P* < .001. Abbreviation: BMI, body mass index.

## Discussion

As hypothesized, results suggested that as children age their ability to report a reasonable or accurate estimate of their height and weight improves. Children underestimate their weight, leading to erroneous obesity prevalence estimates, and the magnitude of underestimation increases with the degree of adiposity. Our findings demonstrate that children exhibit patterns similar to those found in adults regarding self-reported weight. However, our results also indicate that children younger than fifth grade cannot accurately self-report their weight or height. The inability of younger children to accurately self-report height and weight may be due to factors such as perceptual limitations (9), social desirability (3,10), socioeconomic status (11), or exposure to overweight and obesity, which may lead to misperceptions of weight status (12). Future studies should focus on the potential effect of such variables on children’s self-report accuracy. The methods used to collect self-reported and measured height and weight values may have reduced the accuracy of the findings; however, these challenges are consistent with the limitations of conducting school-based research. Regardless, correlations between verbal and written self-report in fifth-graders (0.87, height; 0.99, weight) suggest that the negative effects of our methods were inconsequential. Strengths of this study include the large sample size and cross-section of ages not found in other literature on self-report. Although using self-reported data is desirable because of the low cost, ease of data collection, and the ability to obtain data from a large number of people, children’s inability to report reasonable or accurate values for both height and weight renders self-report an unreliable method for determining BMI and, thus, the prevalence of overweight and obesity among children. These findings indicate the need for educational interventions to improve children’s knowledge and awareness of their height and weight.

## References

[1] Engstrom JL , Paterson SA , Doherty A , Trabulsi M , Speer KL . Accuracy of self-reported height and weight in women: an integrative review of the literature. J Midwifery Womens Health 2003;48(5):338-45. 10.1016/S1526-9523(03)00281-2 14526347

[2] Nawaz H , Chan W , Abdulrahman M , Larson D , Katz DL . Self-reported weight and height: implications for obesity research. Am J Prev Med 2001;20(4):294-8. 10.1016/S0749-3797(01)00293-8 11331120

[3] Rowland ML . Self-reported weight and height. Am J Clin Nutr 1990;52(6):1125-33. 2239790 10.1093/ajcn/52.6.1125

[4] Seghers J , Claessens AL . Bias in self-reported height and weight in preadolescents. J Pediatr 2010;157(6):911-6. 10.1016/j.jpeds.2010.06.038 20688341

[5] Tokmakidis SP , Christodoulos AD , Mantzouranis NI . Validity of self-reported anthropometric values used to assess body mass index and estimate obesity in Greek school children. J Adolesc Health 2007;40(4):305-10. 10.1016/j.jadohealth.2006.10.001 17367722

[6] Himes JH . Can children accurately report their own heights and weights, and why should we care? J Pediatr 2010;157(6):876-8. 10.1016/j.jpeds.2010.07.062 20850764

[7] A SAS program for the CDC growth charts. Centers for Disease Control and Prevention; 2011. http://www.cdc.gov/nccdphp/dnpao/growthcharts/resources/sas.htm.

[8] CDC. Basics about childhood obesity. Centers for Disease Control and Prevention; 2011. http://www.cdc.gov/obesity/childhood/basics.html.

[9] Babooram M , Mullan BA , Sharpe L . Children’s perceptions of obesity as explained by the common sense model of illness representation. Br Food J 2011;113(2):234-47. 10.1108/00070701111105321

[10] Villanueva EV . The validity of self-reported weight in US adults: a population based cross-sectional study. BMC Public Health 2001;1:11. 11716792 10.1186/1471-2458-1-11 PMC59896

[11] Adams K , Sargent RG , Thompson SH , Richter D , Corwin SJ , Rogan TJ . A study of body weight concerns and weight control practices of 4th and 7th grade adolescents. Ethn Health 2000;5(1):79-94. 10.1080/13557850050007374 10858942

[12] Maximova K , McGrath JJ , Barnett T , O’Loughlin J , Paradis G , Lambert M . Do you see what I see? Weight status misperception and exposure to obesity among children and adolescents. Int J Obes (Lond) 2008;32(6):1008-15. 10.1038/ijo.2008.15 18317474 PMC5760224

